# Humans as intuitive classifiers

**DOI:** 10.3389/fpsyg.2022.1041737

**Published:** 2023-01-12

**Authors:** Ido Erev, Ailie Marx

**Affiliations:** ^1^Faculty of Data and Decisions Sciences, Technion Israel Institute of Technology, Haifa, Israel; ^2^Department of Computer Science, Technion Israel Institute of Technology, Haifa, Israel

**Keywords:** J/DM separation paradox, description-experience gap, wavy recency effect, underweighting of rare events, the RUB assumption

## Abstract

Mainstream decision research rests on two implicit working assumptions, inspired by subjective expected utility theory. The first assumes that the underlying processes can be separated into judgment and decision-making stages without affecting their outcomes. The second assumes that in properly run experiments, the presentation of a complete description of the incentive structure replaces the judgment stage (and eliminates the impact of past experiences that can only affect judgment). While these working assumptions seem reasonable and harmless, the current paper suggests that they impair the derivation of useful predictions. The negative effect of the separation assumption is clarified by the predicted impact of rare events. Studies that separate judgment from decision making document oversensitivity to rare events, but without the separation people exhibit the opposite bias. The negative effects of the assumed impact of description include masking the large and predictable effect of past experiences on the way people use descriptions. We propose that the cognitive processes that underlie decision making are more similar to machine learning classification algorithms than to a two-stage probability judgment and utility weighting process. Our analysis suggests that clear insights can be obtained even when the number of feasible classes is very large, and the effort to list the rules that best describe behavior in each class is of limited value.

## Introduction

Classical studies of human decision making (Allais, 1953; Tversky and Kahneman, 1974; Kahneman and Tversky, 1979) use Savage’s (1954) Subjective Expected Utility (SEU) theory as a benchmark. The most influential experimental studies focus on deviations from this benchmark, and the leading descriptive models focus on additions to this benchmark theory that explain the results. This research relies on two implicit working assumptions that facilitate the formulation of clear testable predictions from Savage’s theory. The first implies that the underlying processes can be separated into two distinct stages: Judgment and Decision-Making (Edwards, 1954). Under this “J/DM separation” assumption (Erev and Plonsky, 2022), the decision makers first form beliefs concerning the payoff distributions of the feasible actions, and then use these beliefs (often referred to as judgements) to make decisions. The second assumption is that the participants in properly run experiments Read, Understand and Believe (RUB) the instructions (Erev, 2020).

While Savage’s theory has lost popularity, the two working assumptions that were introduced to facilitate evaluation of this theory still underlie mainstream decision research. The current paper describes some of the negative impacts of this “working assumptions inertia,” and highlights the potential benefit of relaxing these assumptions. Under the proposed relaxation, the cognitive processes that underlie decision making resemble machine learning classification algorithms.

## J/DM separation: The assumption and the paradox

Savage (1954) showed that under a reasonable set of axioms (which generalizes the set used by von Neumann and Morgenstern, 1947 to support Expected Utility Theory), people behave “as-if” they form beliefs concerning the payoff distributions associated with all the feasible actions, and select the action that maximizes personal (subjective) expected utility given these beliefs. To illustrate the potential generality of this theory, Savage describes the preparation of an omelet. Specifically, he considers the decision made after breaking five good eggs into a bowl, and when considering the option of adding a sixth egg. It is easy to see that even this trivial decision is affected by personal beliefs: the belief concerning the probability that the egg is rotten. In addition, the omelet example clarifies the term “as-if” in Savage’s analysis: our experience with the preparation of omelets suggests that it is possible to behave “as-if” we hold beliefs without explicitly considering these beliefs.

**Figure 1:**
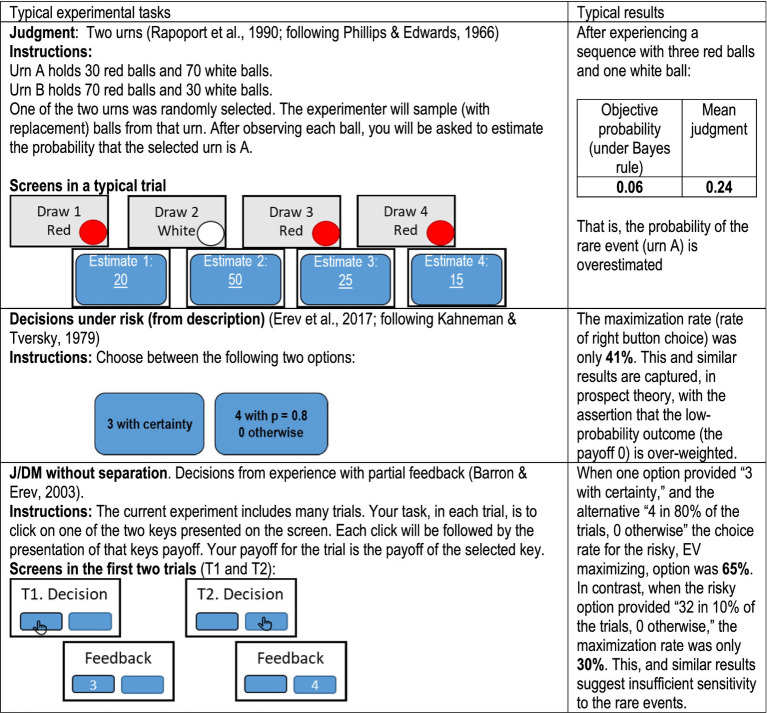
Examples of studies of judgement and decision making with and without the J/DM separation.

As noted above, behavioral decision research focuses on a sequential interpretation of Savage’s theory. Specifically, the “as-if” part is replaced with the assumption that the underlying process can be separated into two stages: Explicit belief formation that involves probability judgment, and decision making. The leading studies of belief formation focus on human judgment; they examine how people estimate the probabilities of different events based on their past experiences. The top panel in Figure 1 presents one example from Rapoport et al.’s (1990) replication of Phillips and Edwards' (1966) classical study of revision of opinion. This study focuses on the way people form beliefs (judge probabilities) based on observable past experiences (the observed draws of red or white balls). The most influential studies of decision-making focus on “decisions under risk,” and explore the way people decide when they are presented with a description of the payoff distributions (and do not have to judge probabilities based on past experience). The middle panel in Figure 1 presents one example from Erev et al.’s (2017) replication of Kahneman and Tversky’s (1979) classical analysis of decisions under risk.

Although separating studies of judgment and of decision making is consistent with a feasible cognitive interpretation of SEU theory, the results presented by Barron and Erev (2003; lower panel of Figure 1) suggest that it can lead to incorrect conclusions. The clearest demonstration of the shortcoming of the J/DM separation comes from studies of the impact of rare (low probability) events. Studies of judgment highlight robust overestimation of the probability of rare events (Phillips and Edwards, 1966; Erev et al., 1994), and studies of decisions under risk document overweighting of low probability outcomes (Kahneman and Tversky, 1979), thus, it is natural to conclude that oversensitivity to rare events is a general tendency (Fox and Tversky, 1998). In sharp contrast to this natural conclusion, Barron and Erev find that in tasks where judgment and decision making are not separated and people decide based on past experiences (as in Savage’s omelet example), their behavior reflects underweighting of rare events. That is, separately both judgment and decision making reflect oversensitivity to rare events, but without the experimental separation these processes often lead to the opposite bias. Erev and Plonsky (2022) refer to this puzzle as the *J/DM separation paradox*.

### The mere-presentation explanation

The difference between the middle and lower panels in Figure 1 is known as the description-experience gap (Hertwig and Erev, 2009): It implies higher sensitivity to rare events in decisions from description (middle panel) than in decisions from experience (lower panel). Erev et al. (2008a) show that part of this gap can be explained as a reflection of a mere-presentation effect: The rare outcomes receive more weight when they are explicitly presented (in the middle panel, but not in the lower panel). Erev and Plonsky (2022) note that the mere-presentation effect can also explain why the deviations from the rational model in judgment from experience (upper panel in Figure 1) are more similar to decisions from description than to decisions from experience. The results suggest that the mere-presentation of the rare events increases their weighting, in both judgment and decision tasks.

The overestimation of the probability of the less likely events in the top panel of Figure 1 can also be explained as the impact of response errors given the bounded response scale (see Erev et al., 1994); since the response scale is bounded between 0 and 1, response errors (e.g., some random responses) are expected to move the mean response toward 0.5. In agreement with this explanation, studies of judgment from experience in tasks in which the bias implied by random responses is minimized (like judgment of the mean of a series of observations, Spencer, 1961) reveal smaller biases (Peterson and Beach, 1967; Lejarraga and Hertwig, 2021). Yet, controlling the impact of response errors does not eliminate the indication of the mere presentation effect in judgment tasks. An indication of the impact of mere presentation that cannot be explained by response error is presented by Fischhoff et al. (1978). In one of the conditions they examined, the participants were asked to judge the probability that the reason for the observation that a “a car will not start,” is “fuel system defective.” The mere-presentation of a list of possible fuel system problems increased the mean estimate from 0.15 to 0.23.

**Figure 2:**
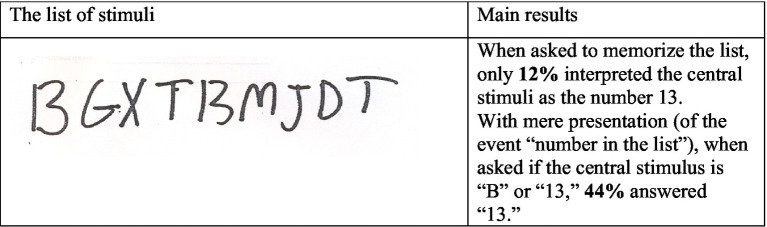
The list of stimuli used by Erev et al. (2008b).

Another indication of the descriptive value of the mere-presentation effect comes from studies that compare implicit and explicit perceptual decisions. One example (from Erev et al., 2008b) is presented in Figure 2. Condition Memory requires an implicit judgment of the probability that the central stimulus is the letter “B” rather than the number “13.” In Condition Memory and Decision, the participants were explicitly asked to decide if the central stimulus is “B” or “13” in addition to being asked to memorize the list. This explicit request includes a presentation of the possibility that the list of letters includes a number. The results reveal that it increased the proportion of participants that remember “13” from 12 to 44%.

## The RUB assumption and the impact of experience

The predictions of SEU theory depend on the information the decision maker uses to form beliefs and decide. Almost any behavior can be consistent with SEU theory given certain assumptions concerning the information the decision maker uses. Thus, it is impossible to test this theory without the addition of auxiliary assumptions regarding that information. The common additions rely on the working assumption that the participants in experimental studies Read, Understand and Believe (RUB) the information provided by the experimenter.

**Figure 3:**
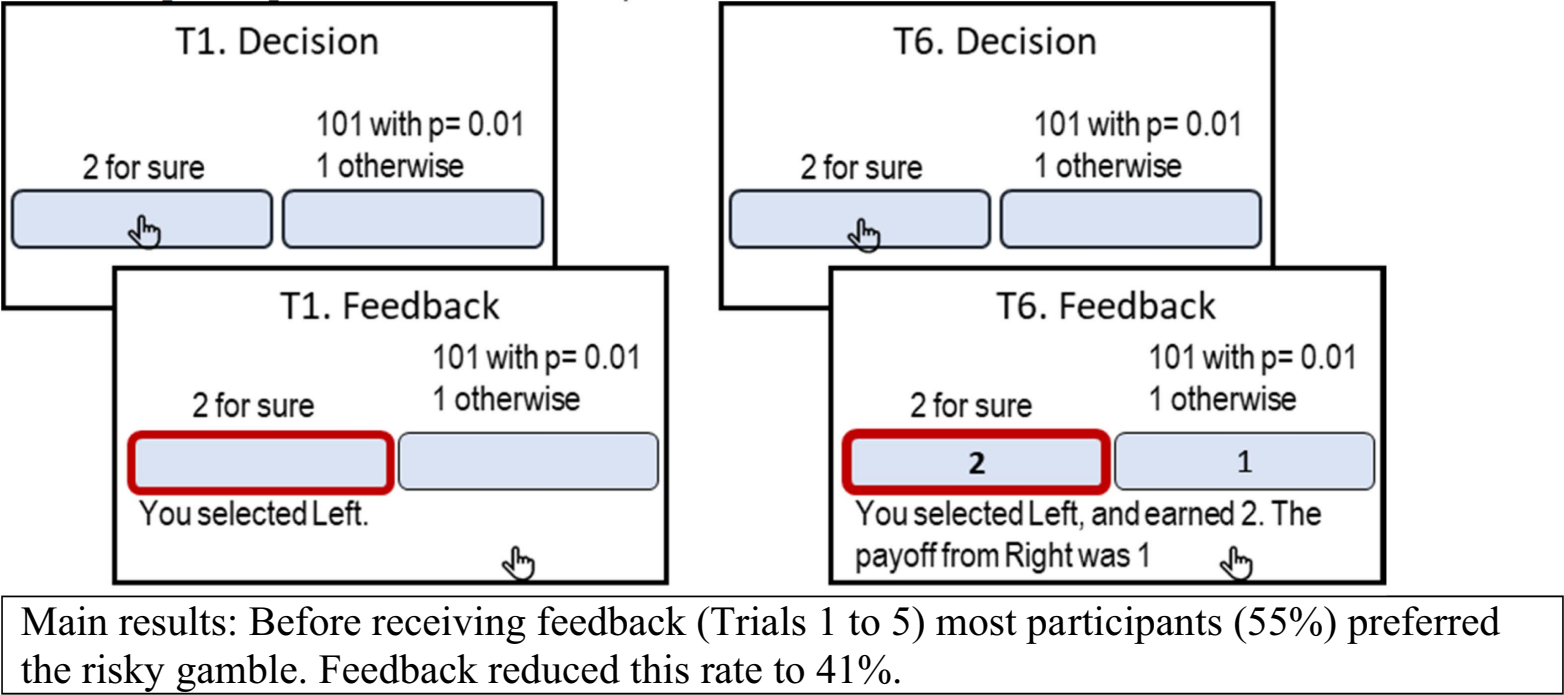
The screens in Trials 1 and 6 in one of the conditions studied by Erev et al. (2017), when the participant chose the left key.

Careful experimenters focus on conditions that facilitate the descriptive value of the RUB assumption, and ensure that rational individuals who RUB the information provided by the experimenter will not be motivated to use other sources of information. For example, careful experimenters use easy to understand instructions, exclude participants that fail attention tests, and avoid running experiments that involve deception. Under these conditions, the RUB assumption implies that the availability of the description of the incentive structure replaces the judgment stage, and determines the information used by the decision makers. However, experimental studies question the success of this effort. For example, in the studies conducted by Erev et al. (2017, and see Figure 3), each of the participants were presented with 30 choice tasks for 25 trials (and were paid for one, randomly selected, of the 750 choices). The participants were first presented with a description of the payoff distributions, and after the 5^th^ trial, received feedback after each choice. The results reveal that the availability of feedback affected the choice rate even when it did not add information concerning the incentive structure. For example, consider the choice task presented in Figure 3, where the participants are asked to select between “2 with certainty” and “1% chance to win 101, 1 otherwise,” 25 times, and told that they will be paid for one randomly selected choice. Erev et al. found that in most cases (55%) the participants chose the risky prospect in the first five trials, but after receiving feedback the choice rate of this prospect dropped to 41%.^1^

## Three direct costs of the J/DM separation and RUB assumptions

In order to clarify the potential negative effects of the tendency to rely on the J/DM separation and the RUB assumptions, and ignore the shortcomings of these assumptions summarized above, we chose to highlight three direct costs of this “working assumptions inertia.”

### Incorrect implementation of basic research results

One of the clearest direct costs of the reliance on the J/DM separation and RUB assumptions is overgeneralization of the results of studies of one-shot decisions under risk (like the middle panel in Figure 1). This research demonstrates overweighting of low probability outcomes. For example, 83% of the participants in Kahneman and Tversky’s (1979) study preferred a loss of 5 with certainty over a 1/1000 chance to lose 5,000. Natural generalization of this finding suggests that the best way to avoid crime involves the use of severe punishments, even if the increase in severity implies lower probability of enforcement. While this prediction seems reasonable under the assumption that people overestimate and overweight rare costs, empirical research shows that using gentle punishments with high probability tend to be more effective (Erev et al., 2010c; Teodorescu et al., 2021). For example, Erev et al. found that asking proctors in college exams to delay the preparation of a map of the students seating (that can be used to detect cheating and justify harsh punishments), and focus on moving students that appear to look around to the first row (a punishment that implies a loss of time of about a minute), reduces cheating.

Another example involves the effort to use lotteries to facilitate COVID-19 vaccination. The use of lotteries is predicted to be effective if people overweight rare rewards, but the effort to use this method to facilitate vaccination was not successful (see Gandhi et al., 2021). In contrast, the use of Green Pass policies that impose gentle punishments on individual that delay vaccination (the requirement to perform time consuming tests to allow entering public areas) appears to be more effective (Mills and Rüttenauer, 2022).

### Suboptimal design of field experiments

In theory, the risk of overgeneralizing basic research can be addressed by running field experiments than compare alternative generalizations. This method is often used by applied behavioral economists that study nudge-based intervention (Thaler and Sunstein, 2008). However, most of these studies focus on the initial reaction to the intervention (see Beshears and Kosowsky, 2020). While this solution is likely to hold if experience does not affect choice behavior, as expected in many settings under the RUB assumption, it might lead to incorrect conclusions if this working assumption does not hold.

### Oversimplification and exaggeration of the impact of the choice environment

One of the contributors to the popularity of the J/DM separation and the RUB assumptions is the fact that they facilitate the simplification of complex decision problems. Yet, in some settings these assumptions simplify the problems too much. One demonstration of the cost of oversimplification is provided by the leading explanations of deviations from maximization in natural settings. Consider risk attitude in financial decisions: The observation that many investors prefer bonds over riskier stocks that provide higher average returns suggests risk aversion (Mehra and Prescott, 1985). In contrast, the observation that investors prefer individual stocks over safer index funds suggests risk-seeking (Statman, 2004). The leading explanations of these contradictories rest on the J/DM separation assumption, and ignore the impact of experience. They imply that the contradictory preferences reflect two distinct biases: Loss aversion in decisions under risk (Benartzi and Thaler, 1995), and overconfidence in probability judgment (Odean, 1998). These explanations suggest that the relative importance of the two biases is a function of the choice environment: Loss aversion is more important when investors choose between stock and bonds (Benartzi and Thaler, 1995), and overconfidence is more important when the investors select between stocks and index funds (Odean, 1998).

**Figure 4:**
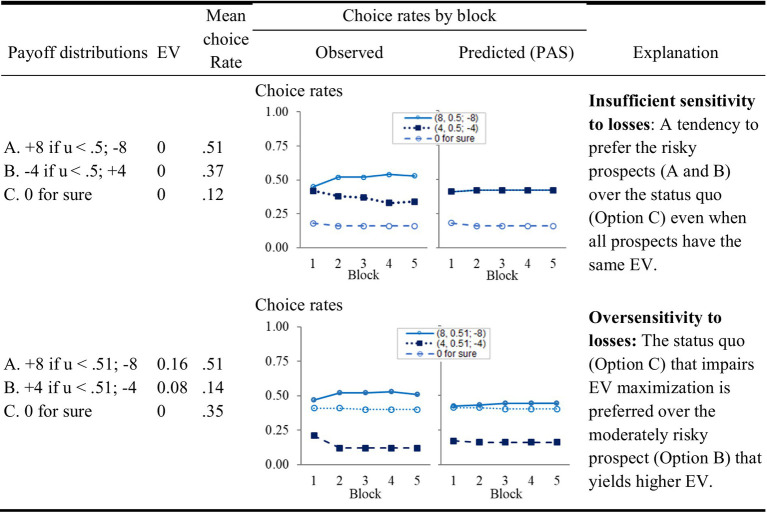
The impact of experience on sensitivity to losses (from Erev et al., 2023). The experiment used a variant of the experimental paradigm described in the lower panel of Figure 1. It included 100 trials, and the participants were presented with the payoff from all options after each choice. The left-hand column presents the incentive structure, u is a random draw from the range 0 to 1 [that is, from u(0,1)]. The right-hand choice rate graphs present the prediction of the PAS model, described below.

Recent research demonstrates that when the impact of experience is considered, the apparent contradiction can be explained without assuming two distinct biases and sensitivity to the choice environment. Specifically, under the assumption that people rely on past experiences, the tendency to select the riskier prospects is highly sensitive to the correlation between the different options. A tendency to avoid the risky options is expected when the differences between the payoffs of these options and the payoff from the safe choice are positively correlated (as in the case of a choice between different stocks and a safe bond), and a tendency to prefer the riskier options is expected these differences are negatively correlated (as in the case of a choice between stocks and index funds, see Ben Zion et al., 2010). Figure 4 presents an experiment (from Erev et al., 2023) that tests and clarifies this prediction. In each of the 100 trials of this experiment the participants were asked to choose between an option that maintained the safe status quo (Option C, “0 for sure”), and two risky options with similar expected return. In the condition summarized in the top left panel, the two risky prospects where *negatively* correlated, and, the choice rate of the status quo was only 12%. In the condition summarized in the bottom left, the two risky prospects where *positively* correlated, and, the choice rate of the status quo was higher (34%) than the choice rate of the more attractive medium risk option (Option B, choice rate of 14%).

## The reliance on small samples assumption, and the intuitive classifier explanation

Previous research that compares alternative explanations of the results exemplified in Figures 3, 4 highlights the advantage of models assuming the people tend to rely on small samples of past experience. Models that share this assumption won four choice prediction competitions (Erev et al., 2010a,b, 2017; Plonsky et al., 2019). The right side of Figure 4 demonstrates how a 2-parameter model of this type captures the contradictory sensitivity to losses described above. The model, referred to as Partially Attentive Sampler (PAS, Erev et al., 2023), assumes that after gaining experience each of the decisions of agent i in Task T is based on a sample of κ_i,T_ past experiences (randomly drawn with replacement) with this task. The value of κ_i,T_ is a free parameter. The agent selects the option with the highest average payoff in the sample, among the options it considers. At each trial the agent considers at least one option. The probability of considering each of the other options equals $1-\delta_{i,T}^{\left[\frac{t-1}{t}\right]}$ , where *δ*_i,T_ is another free parameter. The right-hand column in Figure 4 presents the prediction of this model for Figure 4’s tasks (when the distribution of parameters is estimated on a different set of tasks and different group of participants).

### The wavy recency effect (a violation of the positive recency explanation)

**Figure 5:**
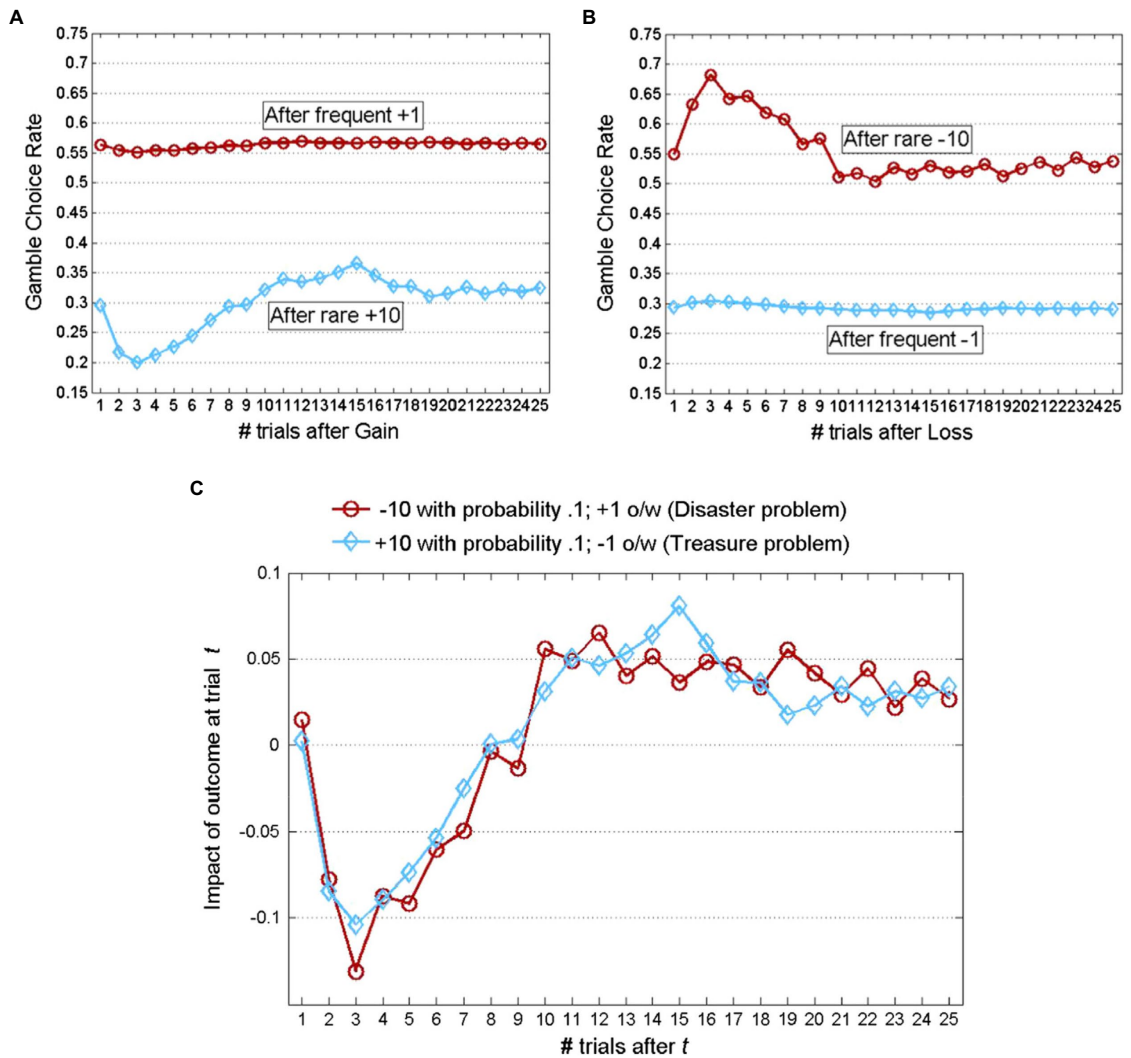
Demonstration of the wavy recency effect (adapted from Plonsky and Erev, 2017). Participants selected repeatedly for 100 trials between two unmarked buttons and received feedback concerning the payoff from both the chosen and the forgone option following each trial. One option generated a payoff of 0 with certainty while the other was a risky gamble detailed in the legend. **(A)** Exhibits the choice rates of the gamble contingent on the gamble providing a gain at trial t; **(B)** exhibits the choice rates of the gamble contingent on the gamble providing a loss at trial t; and **(C)** presents the difference between the corresponding plots in **(A,B)**. Thus, the wavy curves in **(C)** reflect the impact of an outcome generated by the gamble at trial t on its choice rate in subsequent trials. Positive values (on the Y-axis) imply “positive recency” and negative values imply “negative recency.” Data is averaged across 48 participants from Nevo and Erev (2012) and 80 participants from Teoderescu et al. (2013).

The simplest explanations for the predictive value of models that assume reliance on small samples suggest that it reflects cognitive costs and limitations (see Hertwig and Pleskac, 2010). For example, it is possible that people overweight the easier to remember recent trails, or use a simple “win-stay-lose-shift” heuristic (Nowak and Sigmund, 1993). However, analysis of the sequential dependencies in the data rejects this simple explanation (Plonsky et al., 2015). The clearest evidence against the positive recency explanation comes from studies of decisions made between a safe prospect, and a binary risky prospect with a low probability extreme outcome. The results (see typical findings in Figure 5) reveal a wavy recency effect: The tendency to select the best reply to each occurrence of the rare and extreme outcomes is maximal 11 to 16 trials later. Moreover, the lowest best reply rate was observed 3 trials after the occurrence of the rare, extreme outcome.

### The intuitive classifiers explanation

Plonsky et al. show that the wavy recency effect, and the descriptive value of the reliance on small samples hypothesis, can be explained with models that share two assumptions: (1) People try to select the option that led to the best outcomes in the most similar past experiences, and (2) The features used to judge similarity include the sequences of recent outcomes. These assumptions imply that the negative recency part of the wavy recency curve (the drop below 0 in Figure 5C) reflects the fact that the number of “similar past experiences” to decisions made immediately after a sequence that includes rare outcomes tends to be small. Table 1 presents examples that clarify this assertion by focusing of the decision in Trial 64 of an experiment that studies the disaster problem of Figure 5. It shows that if the payoff sequence immediately before Trial 64 includes a rare unattractive outcome (loss of −10), agents that select the option that led to the best outcome after a similar sequence are likely to rely on less than 5 past experiences, and are likely to underweight the rare events. Yet, if the sequence of last three recent payoffs does not include a loss, these agents rely on a larger sample (about 44 observations), and are not likely to underweight the rare events.

**Table 1:** Demonstration of the implications of sequence-based similarity rules.

Trials since the last loss	The payoff from the risky option in the three trials before Trial 64			Expected number of similar past experiences in Trial 64	The probability that the average payoff from the risky option over the similar past experiences is positive (and the implied decision reflects underweighting of rare events)
	Trial 61	Trial 62	Trial 63		
More than 3	+1	+1	+1	44.00	0.495
3	−10	+1	+1	4.70	0.593
2	+1	−10	+1	4.79	0.591
1	+1	+1	−10	4.79	0.602

The table considers Trial 64 in the “disaster problem” of Figure 5 (“0 with certainty” or “10% to lose 10, gain of 1 otherwise), assuming that similarity is determined by the three recent payoffs from the risky option. It shows that when the recent payoff sequence includes a rare event, the number of similar past experiences decreases, and the probability of underweighting of the rare event (choosing the risky option) increases.

**Figure 6:**
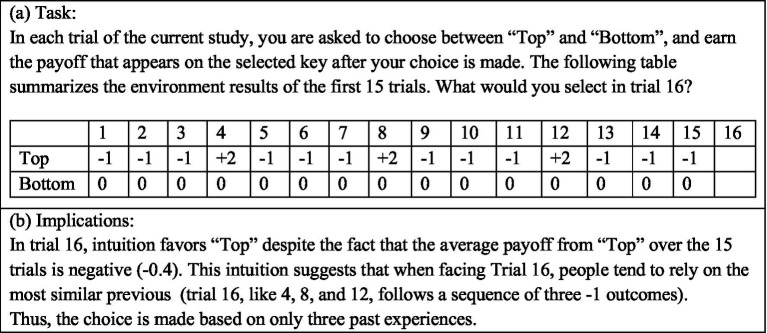
A thought experiment.

**Figure 7:**
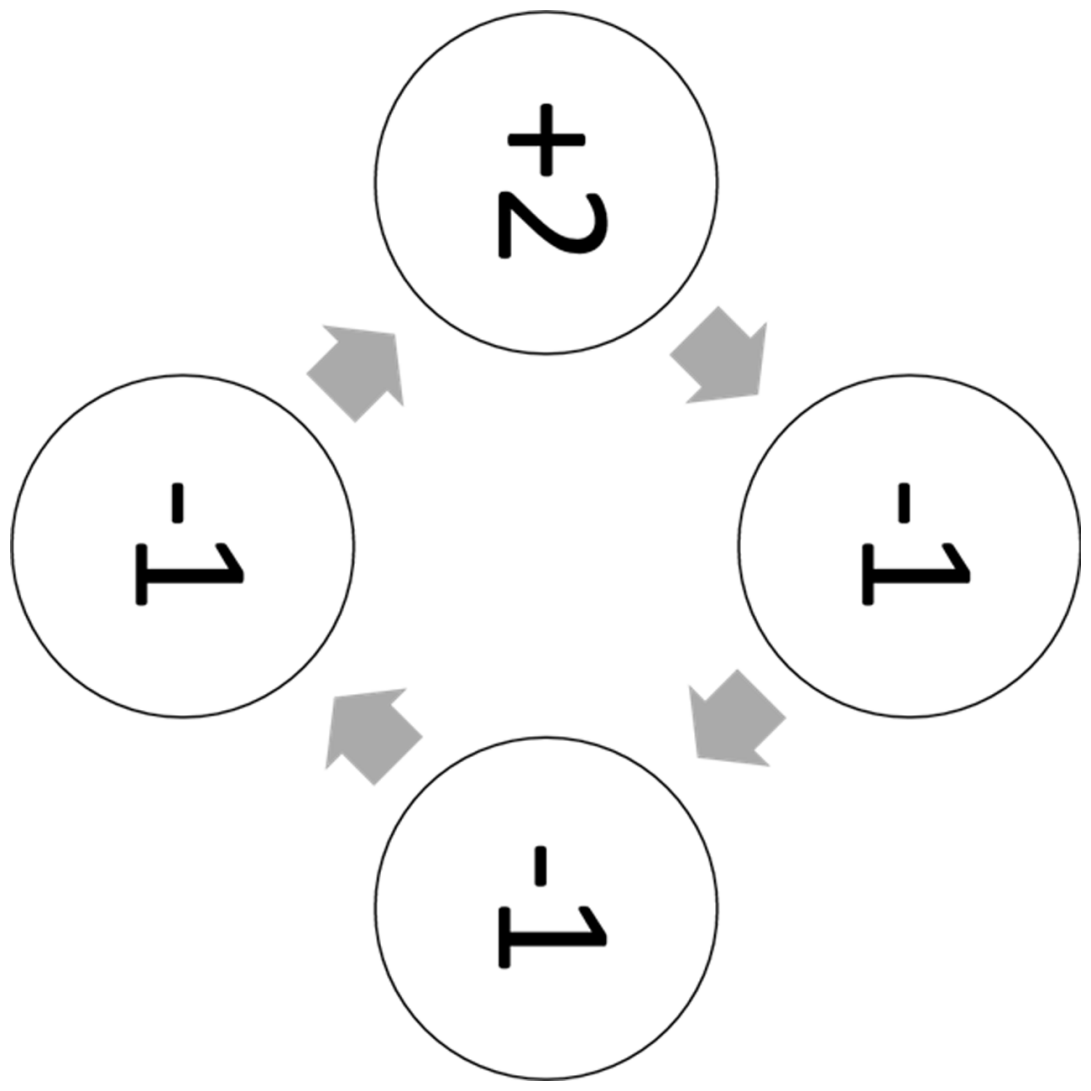
An example of a 4-state Markov chain that could determine the payoff from Top in Figure 6.

Plonsky et al. also demonstrate that when the environment is dynamic, judging similarity based on the sequence of recent outcomes can be highly adaptive. For example, consider the thought experiment described in Figure 6. Intuition in this experiment favors a choice of Top in Trial 16. This behavior is implied by the assumption that similarity is determined based on the number of rare and extreme outcomes in the most recent 3 payoffs. And, under the assumption that the environment is dynamic (e.g., the payoffs are determined by the 4-state Markov chain described in Figure 7) it approximates the optimal strategy.

The assumption that people rely on similar past experiences can also explain the mere presentation effect. The mere presentation of a rare event (e.g., explicit description of the possibility of existence of a letter in a list of digits), under this account, changes the set of experiences that seem most similar to the current task. Specifically, it increases the probability of considering experiences with similar rare events. This account can also capture this initial tendency to overweight rare events in decisions from description (see Marchiori et al., 2015).

**Figure 8:**
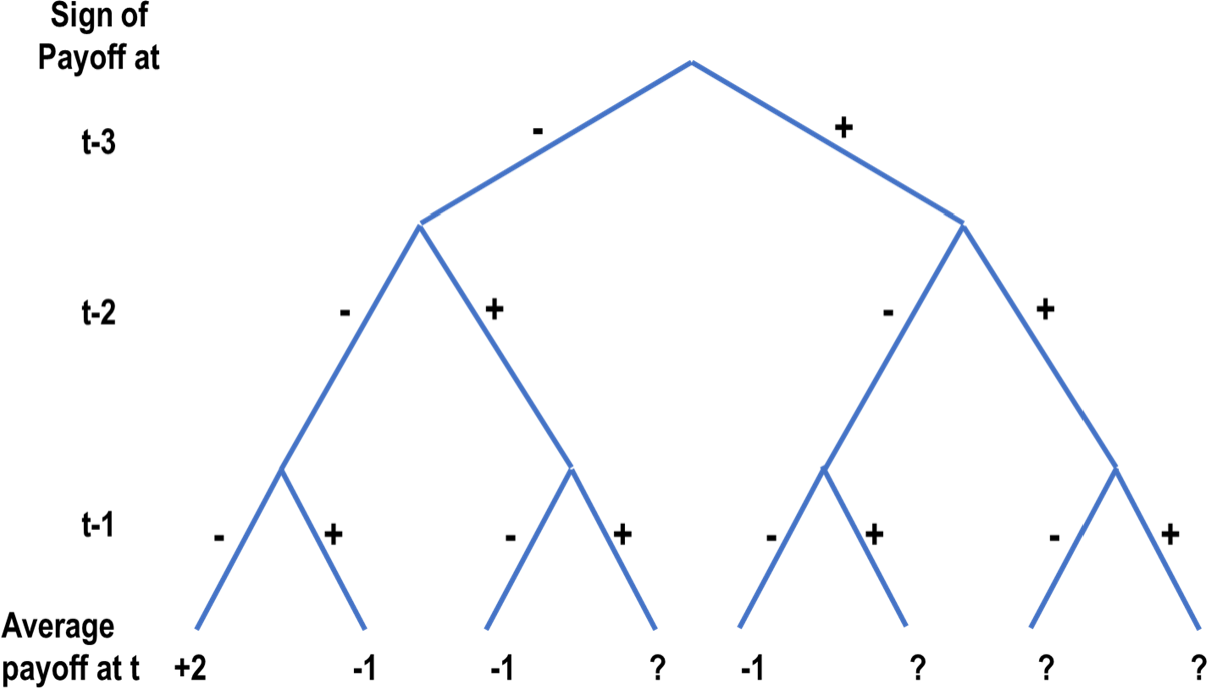
A decision tree analysis of the results in the first 15 trials of the thought experiment presented in Figure 6. The average payoff line presents the observed average payoff in each category. The question mark (?) implies that the training data do not include observation in the relevant branch.

Notice that the current explanation, of the mere presentation effect and descriptive value of the reliance on small samples hypothesis, implies that the underlying processes resemble machine learning classification algorithms like Decision Tree (Safavian and Landgrebe, 1991), and Random Forest (Breiman, 2001). The basic idea behind these algorithms is the classification of the training data based on distinct features, assigning tasks to their appropriate classes, and deriving predictions based on past outcomes in these classes. For example, Figure 8 presents a Decision Tree classification of Figure 6’s 15 observations based on the sign of the payoff from the risky choice in the last three trials (each as an individual feature). Trial 16 in this thought experiment is classified to the left most branch, and the implied decision is Top. While the popular machine learning tools were not designed to capture human cognition, their success (for example, in controlling autonomous vehicles) suggests that it is possible that human cognitive processes were evolved to use the value of effective classifications, and people are “intuitive classifiers.”

It is important to emphasize that the intuitive classifiers explanation is not suggested here as a theory with testable predictions. Moreover, the intuitive classifiers explanation does not imply violations of SEU. Rather, it is an explanation of the observations described above. This explanation can be useful in two ways. First, if highlights the boundary conditions for the predictive value of the models we considered. For example, it implies that models like PAS that assume reliance on random samples of past experiences, and were found to provide good prediction of behavior in static settings, are not likely to provide useful prediction of behavior in dynamic settings (like Figure 7 incentive structure). Second, it sheds light on the way in which these models can be extended.

The intuitive classifiers explanation (or view) is closely related to the assertion that behavior is selected by the contingencies of reinforcement (Skinner, 1985, and see related ideas in Nosofsky, 1984; Gilboa and Schmeidler, 1995; Gentner and Markman, 1997; Dougherty et al., 1999; Marchiori et al., 2015). The current paper contributes to these analyses in two ways. First, the machine learning analogy highlights the possibility that the underlying processes use multiple classification methods, and it may not be possible to develop a simple model capturing people’s response to the contingencies of reinforcements. Second, our analysis demonstrates that when it is difficult to correctly classify the current decision task (the contingencies of reinforcement are not clear) this process is likely to trigger behavior that appears to rely on randomly selected small samples of past experiences. This addition allows useful quantitative prediction of choice behavior in a wide set of situations.

The intuitive classifiers view can also be described as a generalization of the intuitive statistician assertion (Peterson and Beach, 1967; Gigerenzer and Murray, 1987; Juslin et al., 2007). Under the interpretation of the intuitive statistician assertion proposed by Gigerenzer and Murray, people tend to use cognitively efficient rules that approximate the outcomes of the more demanding computation required under traditional statistics. Thus, it assumes that the main deviations from maximization reflect cognitive limitations. The current generalization allows for the possibility of a second type of deviations from maximization: It addresses situations (like the ones considered here) in which the optimal choice rule is simple, but the decision makers cannot know it. In these situations, part of the deviation from maximization appears to reflect the use of cognitively inefficient similarity-based rules.

## Relationship to the adaptive toolkit approach

The analysis presented by Berg and Gigernezer (2010) suggests that the leading behavioral refinements of SEU (including prospect theory, and other analyses that rest on the J/DM and RUB assumptions), are “as-if” models (like SEU itself); these models do no present a cognitively feasible description of the underlying cognitive processes. To advance toward better understanding of the underlying process, Berg and Gigernezer (and see Gigerenzer and Selten, 2001) propose an adaptive toolkit (or toolbox) approach. This apparoch assumes that people use different “fast and frugal” cognitive tools (heuristics) in different settings (Gigerenzer and Todd, 1999). Thus, to understand choice behavior, it is necessary to map of the contextual variables that impact behavior by determining the boundaries of the different areas in the map, and discover the heuristic people use in each area.

The current intuitive classifiers view is similar to the adaptive toolkit approach in several ways, but there are also important differences between the two approaches. One important similarity involves the fact that both approaches assume that decision making starts with a classification process. The main difference involves the assumed number of classes. The adaptive toolkit (or toolbox) approach rests on the (implicit) optimistic assumption that the number of significant classes (distinct areas in the map) is relatively small. This implies that it is possible to map the space of decision tasks, and identify the heuristics that people tend to use in each area of the map. Partial support for this optimistic hypothesis is provided by studies demonstrating how specific “fast and frugal” heuristics can capture adaptive human behavior in specific settings. For example, the take-the-best heuristic (Gigerenzer and Goldstein, 1996) was found to facilitate performance in decisions based on multiple cues, and the priority heuristic (Brandstätter et al., 2006) was found to capture basic decisions from description. The current intuitive classifiers view is less optimistic. We believe that the number of classes that people consider can be extremely large, and it might not be possible to map them in a useful way. To address this possibility, we build on the premise that in many situations the impact of the multiple classifications can be predicted with simple approximations.

Part of our pessimism, concerning the predictive value of fast and frugal heuristics, reflects the outcomes of the choice prediction competitions conducted by Erev et al. (2017) and Plonsky et al. (2019). These competitions focused on decisions from description (without and with feedback concerning the outcome of the previous choices, using the experimental paradigm describe in Figure 3). Under an optimistic interpretation of Brandstätter et al.’s (2006) results, this class of decision tasks is on the area of the map in which people are expected to use the priority heuristic. The results, did not support this prediction. Rather, the best models in the two completions can be described as quantifications of the intuitive classifiers explanation.

One demonstration of the potential of models that approximate the impact of a huge number of possible classifications, comes from the study of decisions from experience in static settings illustrated in Figure 4. As noted above, the choice rates in these experimental conditions can be captured with simple models that assume reliance on small samples, and this behavior can be the product of intuitive classification.

## Summary

Research can be described as a hike through the land of assumptions in an attempt to find a hill with a good point of view on the lands of behaviors (Erev, 2020). Mainstream decision researchers tend to hike on a hill defined by the J/DM separation and RUB working assumptions. The view from this hill clarifies interesting deviations from specific rational models, but can also lead to incorrect conclusions. The current analysis highlights some of the shortcomings of the view from the J/DM separation and RUB hill, and the potential of exploring new areas in the land of assumptions.

The main cost of reliance on the J/DM separation assumption involves incorrect prediction of the impact of rare events. Studies that separate judgment and decision making suggest oversensitivity to rare events, while many natural decisions appear to reflect the opposite bias. This gap can be explained with the assertion that the separation requires an explicit presentation of the rare events that triggers a merge presentation effect. The costs of the RUB assumption include incorrect interpretation of short field experiments, and overestimation of the impact of the choice environment.

The potential of exploring other hills in the land of assumptions is clarified by the high predictive value of models that assume reliance on small samples of past experiences, and the observation that the success of these models can be explained by the assuming that humans are intuitive classifiers. While the intuitive classifiers view does not lead to testable predictions, our analysis suggests that exploring the possibility that people are intuitive classifiers can facilitate understanding and the derivation of models that provide useful predictions.

## Ethics statement

Ethical review and approval was not required for the current study in accordance with the local legislation and institutional requirements. The studies which this study reviews were approved by the Technion IRB committee.

## Author contributions

IE and AM thought about the basic idea together. IE wrote the first draft. All authors contributed to the article and approved the submitted version.

## Funding

This research was supported by a grant from the Israel Science Foundation (grant 861/22).

## Conflict of interest

The authors declare that the research was conducted in the absence of any commercial or financial relationships that could be construed as a potential conflict of interest.

## Publisher’s note

All claims expressed in this article are solely those of the authors and do not necessarily represent those of their affiliated organizations, or those of the publisher, the editors and the reviewers. Any product that may be evaluated in this article, or claim that may be made by its manufacturer, is not guaranteed or endorsed by the publisher.

## Footnotes

^1^Erev et al. also show that the impact of experience cannot be explained by assuming that it only improves understanding of the incentive structure. Their results reveal that experience can increase violations of stochastic dominance. Specifically, when the correlation between the payoffs of the two prospects was negative, experience reduced the choice rate of "50% to win 9, 0 otherwise" over "50% to win 6, 0 otherwise."
